# Dietary cadmium intake and risk of prostate cancer: a Danish prospective cohort study

**DOI:** 10.1186/s12885-015-1153-9

**Published:** 2015-03-26

**Authors:** Kirsten T Eriksen, Jytte Halkjær, Jaymie R Meliker, Jane A McElroy, Mette Sørensen, Anne Tjønneland, Ole Raaschou-Nielsen

**Affiliations:** 1 Danish Cancer Society Research Center, Danish Cancer Society, Strandboulevarden 49, DK 2100 Copenhagen, Denmark; 2 Department of Preventive Medicine and Graduate Program in Public Health, Stony Brook University, New York, USA; 3 Family and Community Medicine, University of Missouri, Columbia, MO USA

**Keywords:** Cadmium, Food, Monitoring, Questionnaire, Cohort, Prostate cancer

## Abstract

**Background:**

Cadmium is classified as a human lung carcinogen based on evidence from high-exposure occupational settings. Though cadmium has no physiological role, increasing evidence suggests cadmium may mimic steroid hormones. This dual ability of being carcinogenic and hormone-like makes cadmium a concern for hormone-related cancers. Causes of prostate cancer are not clear, but steroid hormones, particularly androgens and probably estrogens, may be involved. Cadmium has been positively associated with prostate cancer in occupationally exposed men. In non-occupationally exposed populations, diet and smoking are the main sources of cadmium exposure. The aim of this study was to investigate the association between dietary cadmium intake and prostate cancer risk in Danish men.

**Methods:**

Dietary cadmium intake was estimated in the Danish Diet, Cancer and Health cohort at baseline 1993–97. The estimates were based on a 192 item semi-quantitative food frequency questionnaire and cadmium contents in all food items. Among 26,778 men we identified 1,567 prostate cancer cases from baseline through December 31, 2010 using the Danish Cancer Registry. The association between dietary cadmium intake and prostate cancer risk was analysed using Cox regression models.

**Results:**

We did not find an association between dietary cadmium intake and prostate cancer risk (adjusted incidence rate ratio per 10 μg day^−1^ = 0.98 (95% CI = 0.88-1.10)). The association did not differ according to aggressiveness of prostate cancer. Educational level, smoking status, BMI, zinc or iron intake did not modify the association.

**Conclusions:**

In our study, we did not find an association between dietary cadmium intake and prostate cancer risk in a cohort of Danish men.

## Background

The highly persistent and toxic heavy metal cadmium occurs naturally in the environment. In addition to background levels in soil, cadmium release into the environment relates to fossil fuel combustion, waste combustion and iron and steel production [1,2]. Even in industrially non-polluted areas, farmland may become contaminated by atmospheric deposition and by the use of cadmium-containing fertilizers. Cadmium is used in rechargeable nickel-cadmium batteries, which account for 80% of the world’s production, and in pigments, coatings, stabilizers and alloys [1,3].

Diet is a major source of human exposure to cadmium [3,4]. The highest concentration of cadmium in food is found in shellfish, offal products, and certain seeds, but the main sources of dietary cadmium (around 80%) are cereals, potatoes, root crops, and vegetables. The average cadmium intake from food generally varies between 8 and 25 μg/day [3]. In 2009 the CONTAM Panel of EFSA evaluated the dietary exposure to cadmium in the European population. A tolerable weekly exposure (TWI) value of 2.5 μg/kg bw/week was established. In Denmark, the mean dietary cadmium exposure is estimated at 0.18 μg/kg bw/day, which corresponds to 50% of the TWI value. 5% of the Danish population has an exposure, which exceeds the TWI value [5]. Smoking is another important source of cadmium exposure, since cadmium is able to build up in the tobacco plant. A single cigarette contains 1–2 μg cadmium with approximately 10 percent of the cadmium content inhaled. Smokers typically absorb a similar amount of cadmium to food ingestion (1–3 μg per day) [3].

Cadmium accumulates in the human body and is efficiently retained in the kidney, where it remains for many years (half-life: 10–30 years). Most of the cadmium is bound to metallothionein, an inducible metal-binding protein that functions in the homeostasis of heavy metals (e.g., zinc) and provides protection against many of cadmium’s toxic effects [6]. The iron-cadmium ratio is also important, since low body iron stores are shown to be linked to increased intestinal absorption of cadmium [7].

Cadmium has been classified as a human carcinogen by the International Agency for Research on Cancer (IARC, Lyon, France) based on mechanistic and epidemiologic evidence from high-exposure occupational settings [8]. Suggested mechanisms of cadmium carcinogenesis include oxidative stress, DNA damage, altered DNA repair, enhanced proliferation and/or depressed apoptosis [2,9].

Recent research has shown that both androgens and estrogens may play a role in the development of prostate cancer, e.g., chronic exposure to testosterone and estradiol was strongly carcinogenic for the prostate of rats, whereas testosterone alone was only weakly carcinogenic [10]. Estradiol plus testosterone treatment induces acinar lesions that are similar to human prostatic intraepithelial neoplasia [10]. Studies have shown that cadmium exerts estrogenic activity, including proliferation of breast cancer cells [11-13] and activation of the estrogen receptor-α [12-15]. Cadmium has also shown androgenic activity in which treatment of prostate cells with cadmium stimulated cell growth, increased gene expression and activated the androgen receptor [16]. Also, in castrated animals, a single low dose of cadmium increased the weight of the prostate. These *in vitro* and *in vivo* effects were blocked by anti-androgen [16].

Epidemiological studies do not convincingly indicate that cadmium exposure is a risk factor of prostate cancer. Occupational studies have examined the relationship between high cadmium exposure and prostate cancer risk and many [17-21] but not all [22,23] found cadmium exposure to be a risk factor for prostate cancer. Population-based studies on low cadmium exposure and prostate cancer are overall inconclusive [24-28].

The aggressiveness of prostate cancer can be expressed by prostate cancer stage and grade of the prostate tumor. Localized and low-grade prostate cancer tumors may have different etiologies compared to advanced and high-grade tumors [29].

The aim of this study was to investigate prospectively whether dietary cadmium intake was associated with prostate cancer risk in Danish middle-aged men. We also evaluated whether the association differed by aggressiveness of prostate cancer. Further, we explored potential effect modification by educational level, smoking status, BMI, dietary iron intake or zinc intake.

## Methods

### Study population

From December 1, 1993, through May 31, 1997, 27,178 men and 29,875 women, who were aged 50–65 years, born in Denmark, and had no previous cancer diagnosis, were enrolled in the prospective Diet, Cancer and Health (DCH) cohort [30]. At enrolment, the participants completed a self-administered, interviewer-checked 192 item semi-quantitative food frequency questionnaire and a questionnaire covering lifestyle habits including information on smoking, physical activity, social factors and health status.

This study was approved by the regional research ethic committee for Copenhagen and Frederiksberg, and written informed consent was obtained from all participants.

### Exposure assessment

We estimated the average dietary cadmium intake per day for each person in the prospective DCH cohort based on the 192 item semi-quantitative food frequency questionnaire filled in at enrolment. For the calculations we used food monitoring data from The Danish Food Monitoring Programme for Nutrients and Contaminants, 1993–97 [31]. The Danish Food Monitoring Programme was initiated in 1983 and monitoring cycles run for 5-year periods to allow for a comparison of trace element contents (including cadmium) over time in food items sold in Denmark and to assess the potential health concerns of the dietary intake of the trace elements investigated. The samples of each food item were analysed individually, giving detailed information on the variation of trace elements in food items sold on the Danish market. The number of samples analysed of each specific food item was decided on the basis of earlier experience concerning the variation in contents of trace elements in that specific food item, such that the number of samples analysed were larger for food items with larger variation in trace element concentration than for food items with lesser variation. Cadmium concentrations of the specific food items were averaged. For our study, dietary cadmium measurements from the 5-year monitoring period 1993–97 were used, since this period matches with the period of completion of the food frequency questionnaire in the DCH cohort. The contents of more than 80 different foods were monitored from 1993–97. For food items where data were not available during this period, we used data from the monitoring period 1998–2003, and data from unspecified years. The obtained cadmium concentration for each food item was added to the food table using the FoodCalc program [32] and we obtained an estimate of average dietary cadmium intake per day (μg cadmium per day) for each participant in the DCH cohort.

### Outcome assessment

We used the Danish Cancer Registry, containing accurate and virtually complete data on cancer incidence in Denmark, to identify all cases of prostate cancer cases in the cohort from enrolment to December 31, 2010. Data was made available from Statens Serum Institut, who registers all newly diagnosed cases of cancer in Denmark. Accessing data from the Danish Cancer Registry for the purposes of this study was permitted by the Danish Data Protection Agency. Definition of prostate cancer was based on the 10th Revision of the International Classification of Diseases: C61. To categorize prostate cancer aggressiveness, cases diagnosed up until December 31, 2008 were classified as either aggressive or non-aggressive defined by Gleason score, PSA test results at diagnosis, and TNM. These data were obtained from a thorough review of medical records. Cases with Gleason score ≥7, PSA >15, T-stage ≥3, N-stage ≥1, or M-stage ≥1 were defined as aggressive [33]. For cases where relevant information for classification of aggressive prostate cancer was not clearly apparent at first review, a thorough review by a medical doctor was conducted to obtain the needed information in the record. Non-aggressive prostate cancer cases were defined as those who did have the relevant information for defining aggressiveness of prostate cancer, but who do not meet the criteria of being aggressive.

### Statistical analyses

Cox proportional hazard models were used for statistical analyses. Age was underlying time scale [34], ensuring comparison of individuals of same age. We used left truncation at age of enrolment, so that people were considered at risk from enrolment into the cohort, and right censoring at the age of cancer diagnosis (except non-melanoma skin cancer), death, emigration, or December 31, 2010, whichever came first.

We estimated crude and adjusted incidence rate ratios (IRRs) using the estimate of dietary cadmium intake as a continuous variable. Data on potential confounders were derived from the questionnaires administered at enrolment. The analyses were adjusted for the following *a priori* defined potential confounders: Educational level (<8 years; 8–10 years; >10 years), smoking status (never; former; current), BMI (continuous), waist-to-hip ratio (continuous) and physical activity (MET score, continuous). Linearity was evaluated using linear splines with three boundaries and there was no significant deviation from linearity. Also, we estimated crude and adjusted IRRs by tertiles of daily dietary cadmium intake, based on distribution among the cohort members, using lowest tertile as reference group. We also evaluated *a priori* specified individual characteristics as potential effect modifiers: Educational level (<8, 8-10y, >10y), smoking status (never, former, present), BMI (<25, ≥25), total zinc intake (<median, ≥median), and total iron intake (<median, ≥median). Effect modifications were evaluated by introducing interaction terms into the model, and were tested by the Wald test. Also, we calculated separate IRRs for aggressive and non-aggressive prostate cancer, respectively. For this analysis, 400 cases were excluded due to missing information on aggressiveness status.

For statistical analyses we used the procedure PHREG in SAS version 9.3 (SAS Institute, Cary, North Carolina, USA).

## Results

Among the 27,178 men of the DCH cohort we excluded 234 with a cancer diagnosis before baseline, 1 with unknown month of cancer diagnosis, 30 with no dietary cadmium exposure data, and 135 with incomplete covariate data. This resulted in a study population of 26,778 men (1,567 cases) with complete covariate data. Among the cases, 840 were defined as aggressive cancer cases and 327 were defined as non-aggressive prostate cancer cases. Mean follow-up time was 13 years for the whole study population.

Distributions of relevant baseline characteristics of prostate cancer cases and cohort are shown in Table 1. The mean estimated daily cadmium intake of the cohort was 16 μg per day (5-95% percentiles = 9–25 μg). Similar levels of dietary cadmium intake were observed for the cancer cases and the cohort. The right part of Table 1 shows that the proportion of men with high educational level increased with increasing tertiles of dietary cadmium exposure whereas the proportion of men with low and medium educational level decreased with increasing cadmium exposure. Also, the proportion of never smokers increased with increased dietary cadmium exposure whereas the opposite was seen for current smokers. Higher zinc intake, iron intake and physical activity were all associated with higher dietary cadmium exposure.

**Table 1:** **Baseline Characteristics of Prostate Cancer Cases and Cohort and by Tertiles of Dietary Cadmium Intake of the Cohort in the Diet, Cancer and Health Study, 1993-97**

			Cohort (N = 26,778)		
Baseline Characteristics	Cases (N = 1,567)	Cohort (N = 26,778)	<14 μg Cd/day	14-18 μg Cd/day	>18 μg Cd/day
	*N (%)*				
Educational level					
Low	540 (35)	9,315 (35)	3,266 (37)	3,095 (35)	2,954 (33)
Medium	633 (40)	11,126 (41)	3,872 (43)	3,779 (42)	3,475 (39)
High	394 (25)	6,337 (24)	1,787 (20)	2,053 (23)	2,497 (28)
Smoking					
Never	427 (27)	6,868 (26)	2,115 (24)	2,287 (26)	2,466 (28)
Former	580 (37)	9,282 (34)	2,916 (32)	3,120 (35)	3,246 (36)
Current	560 (36)	10,628 (40)	3,894 (43)	3,520 (39)	3,214 (36)
	*Median (5-95%), mean*		*Median (mean)*		
Age (years)	58 (51–65), 58	56 (51–64), 57	56 (57)	56 (57)	56 (57)
BMI (kg/m^2^)	26 (22–32), 26	26 (21–33), 27	27 (27)	26 (27)	26 (26)
Waist-to-hip ratio	0.95 (0.86-1.06), 0.96	0.95 (0.86-1.06), 0.96	0.96 (0.96)	0.95 (0.96)	0.94 (0.95)
Physical activity (MET score)	55 (16–164), 68	54 (17–150), 65	48 (58)	54 (64)	62 (73)
Zinc intake (mg/day)^a^	17 (11–33), 19	18 (10–33), 20	14 (16)	17 (19)	21 (23)
Iron intake (mg/day)^a^	16 (9–31), 17	16 (9–31), 18	12 (15)	15 (18)	19 (22)
Dietary Cd intake (μg/day)	16 (10–24), 16	16 (9–25), 16	12 (11)	16 (16)	21 (21)

*Abbreviations:BMI* Body mass index, *MET* metabolic equivalent, *Cd* cadmium.

^a^Sum of intake from diet and supplement.

Cereals and vegetables, including potatoes, together contributed to the majority of the estimated dietary cadmium exposure in our study population with a mean of 84% (SD = 7%). Specifically, whole grain cereals contributed a mean of 34% (SD = 13%), potatoes 23% (SD = 11%), vegetables, excluding potatoes, 13% (SD = 7%) and refined cereals 15% (SD = 9%). In contrast, meat (red meat, poultry and processed meat), fish, fruit and dairy products collectively only contributed with a mean of 6.3% (SD = 2.8%) of the mean cadmium intake (data not shown).

We did not find a significant association between dietary cadmium intake and risk of prostate cancer, neither in linear nor categorical analyses (Table 2). Also, no significant incidence rate ratios were observed for neither aggressive nor non-aggressive prostate cancer, when analysing these subtypes separately. Table 3 shows that education, smoking, BMI, zinc and iron intake did not modify the association between cadmium and prostate cancer. There was a weak tendency toward smoking status being an effect modifier, as the subgroups of former smokers showed positive and current smokers showed negative associations, and we did not find any association for never smokers, but the interaction was insignificant.

**Table 2:** **Incidence rate ratios of prostate cancer according to daily dietary cadmium intake**

Study population	Dietary cadmium exposure	N cases	Crude model	Adjusted model^a^
			IRR (95% CI)	IRR (95% CI)
**Total study group**	10 μg increment day^−1^	1567	1.01 (0.90-1.12)	0.98 (0.88-1.10)
	Tertiles:			
	<14 μg day^−1^	516	1.00	1.00
	14-18 μg day^−1^	516	0.97 (0.85-1.10)	0.96 (0.85-1.08)
	>18 μg day^−1^	535	0.99 (0.88-1.12)	0.97 (0.86-1.10)
**Subgroups** ^**b**^				
Aggressive prostate cancer	10 μg increment day^−1^	840	1.02 (0.88-1.17)	1.00 (0.86-1.16)
Non-aggressive prostate cancer	10 μg increment day^−1^	327	1.02 (0.81-1.29)	0.99 (0.77-1.25)

*Abbreviations:IRR* incidence rate ratios, *CI* confidence interval.

Age is underlying time-scale.

^a^Adjusted for educational level (<8 y; 8-10y; >10y), smoking status (never; former; current), BMI (continuous), waist-to-hip ratio (continuous), and physical activity (MET score, continuous).

^b^Classification is based on Gleason score, PSA test results at diagnosis, and TNM. Of the 1,567 cases, 400 were excluded in the analyses due to missing information on prostate cancer aggressiveness.

**Table 3:** **Association between dietary cadmium intake and total prostate cancer by different baseline characteristics**

Stratification factors	N cases	Crude model	*p* ^a^	Adjusted model^b^	*p* ^a^
		IRR (95% CI)		IRR (95% CI)	
Education					
Low (<8y)	540	0.96 (0.80-1.15)		0.95 (0.79-1.14)	
Medium (8-10y)	633	1.01 (0.85-1.20)	0.88	1.00 (0.84-1.19)	0.90
High (>10y)	394	1.03 (0.84-1.26)		1.01 (0.82-1.24)	
Smoking					
Never	428	0.99 (0.81-1.21)		0.97 (0.79-1.18)	
Former	580	1.15 (0.97-1.37)	0.08	1.13 (0.95-1.35)	0.09
Current	561	0.87 (0.72-1.04)		0.86 (0.71-1.03)	
BMI					
<25	1000	0.93 (0.78-1.11)	0.33	0.92 (0.77-1.09)	0.29
≥25	567	1.04 (0.91-1.19)		1.03 (0.90-1.18)	
Total zinc intake					
< median	798	1.04 (0.86-1.24)	0.89	1.00 (0.84-1.21)	0.99
≥ median	769	1.02 (0.88-1.18)		1.00 (0.86-1.16)	
Total iron intake					
< median	798	1.08 (0.89-1.32)	0.59	1.05 (0.86-1.29)	0.67
≥ median	769	1.01 (0.87-1.17)		1.00 (0.86-1.16)	

*Abbreviations:IRR* incidence rate ratio, *CI* confidence interval, *BMI* body mass index.

^a^P values for interaction.

^b^Adjusted for educational level (<8 y; 8-10y; >10y), smoking status (never; former; current), BMI (continuous), waist-to-hip ratio (continuous) and physical activity (MET score, continuous). BMI (continuous) was not including in the stratification analyses on BMI.

The stratification factors are education, smoking, BMI, zinc intake and iron intake. Incidence rate ratios are per 10 μg increment day^−1^.

## Discussion

We found no clear association between dietary cadmium intake and prostate cancer. A previous prospective population-based case–control study evaluated the association between pre-diagnostic toenail cadmium and prostate cancer risk in a cohort [25]. In agreement with our findings, this study did not find an association between cadmium and risk for prostate cancer. Also, a population-based case–control study found no significant association between dietary cadmium intake and prostate cancer among men aged 45–67 regardless of prostate cancer type. Same study found no significant association for aggressive tumors among men aged 68–74, but report a significant association for all prostate tumors comparing the highest quartile of cadmium exposure (but not the second and third quartile) with the lowest for this age group [28]. A small hospital-based case–control study showed higher risk for prostate cancer in association with toenail cadmium levels [27], and a population-based prospective cohort study found an association between dietary cadmium intake and localized prostate cancer but did not find an association for advanced prostate cancer [24]. A recent meta-analysis analyzed the results of eight previous studies that have investigated the association of dietary cadmium intake and cancer risk [35]. Overall, dietary cadmium intake showed no statistically significant association with cancer risk, but subgroup analyses (using study design, geographical location, and cancer type) indicated positive association between dietary cadmium intake and cancer risk among studies conducted in Western countries, particularly with hormone-related cancers, including prostate cancer. However a limited number of studies were included in the meta-analyses, which limits the possibility to draw significant conclusions, especially in the subgroup analyses. Most occupational studies [17-21] though not all [22,23] found cadmium exposure to be a risk factor for prostate cancer. This trend of positive findings among the occupational exposed population studies could potentially be attributed to the higher cadmium exposure level prevailing in these studies.

The zinc-cadmium ratio is generally considered important, as cadmium toxicity and storage are greatly increased with zinc deficiency [36,37]. In our study we also explored the potential effect modification by total zinc intake, but as in another study [24] zinc did not modify the association between cadmium intake and prostate cancer in our study. Another study using toenail cadmium and zinc concentrations found no evidence that the patterns of association between cadmium and prostate cancer differed by concentrations of zinc or vice versa [25] whereas a significant inverse association between cancer mortality and zinc-to-cadmium ratio was found for both genders in yet another study [38]. Low iron stores is linked to a higher intestinal absorption of cadmium, e.g. low iron status as determined by low serum ferritin has been shown to result in significantly higher blood cadmium level [39]. However, in our study iron intake did not modify the association between cadmium and risk of prostate cancer. However, since zinc is suggested to increase the sequestering of cadmium and as an increased absorption of cadmium is associated with reduced body iron stores, the role of cadmium versus zinc intake and iron intake will need to be investigated further in future epidemiologic studies of prostate cancer.

In order to minimize the potential effect of exposure to endogenously produced adipose tissue hormones, we performed analyses stratified by baseline BMI. We expected an association to be easier detected among those with lower BMI, since this group (with lower adipose tissue-derived hormone exposure) have a reduced influence of endogenous hormone exposure. However, we found no statistically significant interaction with BMI.

Cigarette smoking is an equivocal factor in terms of studying hormone-related cancer, as tobacco smoking on the one hand is a significant source of the hormone-mimicking cadmium and on the other hand has some anti-estrogenic properties [40], perhaps masking a cadmium effect. In a recent European cohort study, including data from the present DCH cohort, smoking was found to be associated with a small reduction in the risk of prostate cancer, which was significant for less aggressive prostate cancer [41]. In our study, adjusting for smoking status either with or without other potential confounders did not change the association between dietary cadmium exposure and prostate cancer. Also, including smoking duration and smoking intensity in the adjusted model did not change results. We investigated the potential effect modification by smoking status, and expected a potential association to be easier to detect among never smokers, since this group is not influenced by cadmium exposure from cigarettes. We found a weak tendency toward smoking status being an effect modifier for the association between dietary cadmium and prostate cancer, as the subgroups of former smokers and current smokers showed positive and negative associations, respectively, but we did not find an association for never smokers. These results did not follow the expected pattern with never smokers showing the least influence, followed by former smoker and current smokers showing the strongest influence. This suggests that smoking may play a more complex role in prostate cancer risk. Therefore, results of analyses stratified by smoking status should be interpreted with caution.

It has been proposed that localized and low-grade prostate cancer tumors could have another etiology than advanced and high-grade tumor [29]. In our study, we found no difference in the relationship between dietary cadmium intake and prostate cancer risk by degree of aggressiveness.

A major strength of this study is the prospective design based on a well-defined cohort with potential confounder data available. Virtually complete nationwide registries supplied information on cancer diagnosis and vital status for the cohort. Prostate cancer status could not have biased the exposure assessment because food frequency and lifestyle questionnaires were collected before any diagnosis of cancer. Further, as the disease rarely occurs in men under 50 years of age, our study group is ideal in that respect as the cohort participants were enrolled at the age of 50–64 [30].

This study also has limitations; especially non-differential exposure measurement error in the dietary cadmium estimate could mask a real association with prostate cancer risk. Members of the investigated cohort were asked to report their average dietary habits within the year prior to enrolment, and, accordingly, their answers may not fully reflect long-term dietary pattern. Also, some deviation in the content of cadmium in specific food items could be another source of measurement error. That is, a limitation of this study includes our ability to accurately assess dietary cadmium intake, which may have masked a true association. Finally, dietary cadmium intake assessed using FFQ is not a measure of total cadmium exposure, which also included cadmium exposure from smoking and occupational exposures. However, statistically we were able to adjust for smoking and perform stratification analyses on smoking status.

## Conclusion

We did not find an association between dietary cadmium exposure and prostate cancer risk in Danish middle-aged men. More studies are needed to substantiate our findings.
